# Supplementary material for: JESTR: Joint Embedding Space Technique for Ranking candidate molecules for the annotation of untargeted metabolomics data
Source: Bioinformatics. 2025 Jun 17;41(7):btaf354. doi: 10.1093/bioinformatics/btaf354 (PMC12233093; doi:10.1093/bioinformatics/btaf354)
Supplement: btaf354_Supplementary_Data [file btaf354_supplementary_data.pdf]

JESTR: **J**oint **E**mbedding **S**pace **T**echnique for **R**anking Candidate Molecules for the Annotation of Untargeted Metabolomics Data
Supplementary Information

Apurva Kalia[1], Yan Zhou Chen[1], Dilip Krishnan[2], and Soha Hassoun[1,3]

[1]Department of Computer Science, Tufts University, Medford, MA 02155, USA
[2]Google Research
[3]Department of Chemical and Biological Engineering, Tufts University, Medford, MA 02155, USA.

S1 Batching strategy for contrastive learning and regularization

During contrastive learning, each batch of size k contains k target molecules. In each training epoch, the spectrum corresponding to any given target molecule is chosen sequentially from the list of paired spectra. The batch size k was set to 32. The value of the temperature hyper-parameter τ was set to 0.05. For all experiments, contrastive learning was stopped at 1000 epochs, where it was observed that the contrastive loss stopped reducing significantly. The batch size for the candidates, k_{aug}, was set to 32.

During regularization, for each training molecule in the batch, a set of most similar candidates for that molecule are used to calculate the regularization loss. This is done sequentially for every mini-batch in the epoch. A diagram outlining our batching strategy is shown in Figure S1.

S2 Implementation details for JESTR

JESTR was trained on NVIDIA A100 GPU with 40GB of graphics RAM and 256GB of CPU RAM. Adam (Kingma and Ba, 2014) was used as the optimizer. We used grid search over the ranges of the tuned parameters. The values of the parameters that achieved the best performance were selected and used to train and test the model for all datasets (Table S1).

Table S1: Tuning of various hyperparameters for JESTR model using grid search.

Hyperparameter	Values searched	Final value
Contrastive Learning Rate	5e-6, 5e-5, 5e-4	5e-4
Contrastive Batch Size	32, 64, 128	32
Early Stopping Epochs	20, 40, 80	80
Regularization loss weight	0.1, 0.3, 0.5	0.1
% epochs used for regularization	3, 10, 20	3

We also looked at the training and inference times of all the models (Table S2). The runtimes were measured on a Linux machine with 48 CPU cores with 196GB RAM and 6 nVidia A5000 GPUs with 24GB RAM. JESTR uses a GNN encoder for the molecules and an MLP encoder for the spectra. MIST uses MLP and transformers as its encoders for spectra, while ESP uses MLP and GNN as encoders for spectra and molecules, respectively. From a computational complexity of the model architecture, the three models are similar. The training time depends largely on the amount of training each model undergoes.

Table S2: Training time and inference time for the 3 models on the NPLIB1 dataset. The runtimes were measured on the same machine. JESTR takes longer to train because the training is run for a larger number of epochs.

Model	Training Time	Epochs	Inference Time
JESTR	6 hours	800	1.5 hours
MIST	1.2 hours	50	20 minutes
ESP	1.3 hours	100	1.2 hours

S3 JESTR ranks and target-candidate separation on NIST2020 and MoNA

We look at how JESTR separates target and candidate molecules for different datasets in Figures (S2 and S3)

S4 Regularization analysis for NIST2020 and MoNA

We study the impact of regularization on molecule ranking and on molecule embeddings for all datasets in Figures (S4 and S5).

S5 MCES Distributions between Train and Test Molecules

To fairly benchmark against SIRIUS and CFM-ID's pretrained models, we evaluate the distribution of the MCES distances between the pretrained molecules of SIRIUS and CFM-ID and the test molecules of NPLIB1, NIST2020, MoNA, and MassSpecGym. For each dataset, we randomly select 10% of the train-test pairs and compute the MCES distance. We plot the distributions for SIRIUS in Figure S6 and CFM-ID in Figure S7. The test set in MassSpecGym provides the most equitable setup, where the pretrained molecules are structurally different from the test molecules by an MCES distance of 10 or greater, just as the original MassSpecGym split (Figure S8).

References

Kingma, D. P. and Ba, J. (2014). Adam: A method for stochastic optimization. *arXiv preprint arXiv:1412.6980*.

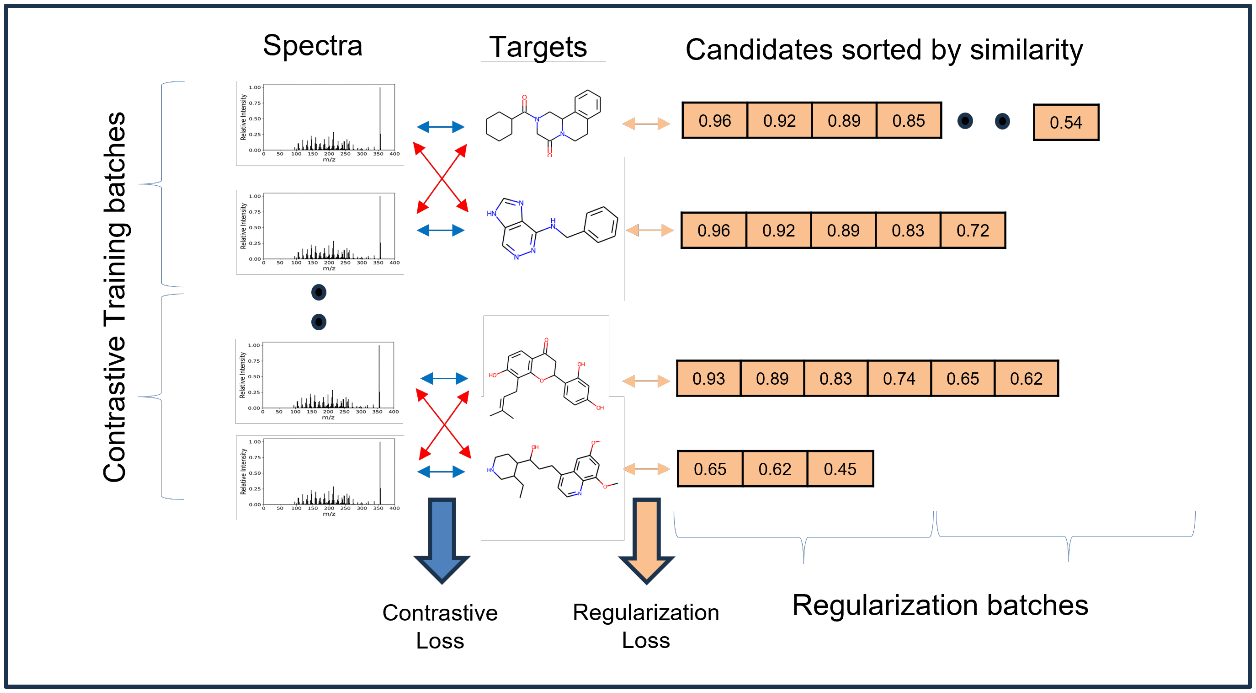

Figure S1: The contrastive loss is calculated over a batch of molecules at a time. For each molecule in the batch, candidates are selected from the candidate list sorted by similarity. If a particular molecule has a lesser number of candidates than the batch size, the candidates for that molecule will be repeated sequentially. The candidates thus selected are used to calculate the regularization loss. The curly braces in the vertical direction show the contrasting batching for two batches, while the curly braces in the horizontal direction show regularization batches for two batches

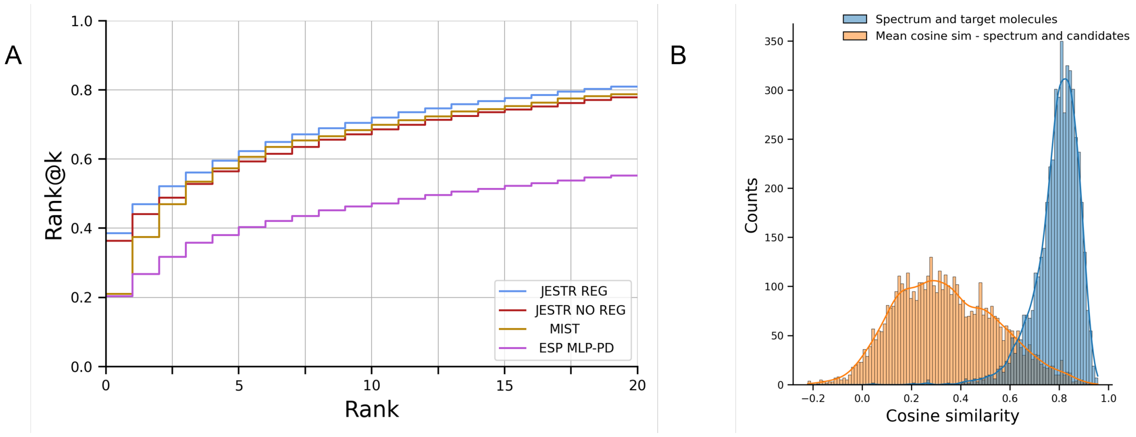

Figure S2: Results. A. Rank@k results for JESTR on NIST2020, with and without regularization, MIST, and ESP MLP-PD. B. Distribution of cosine similarities of query spectra and target/candidate molecules in the NIST2020 test set with contrastive learning using JESTR.

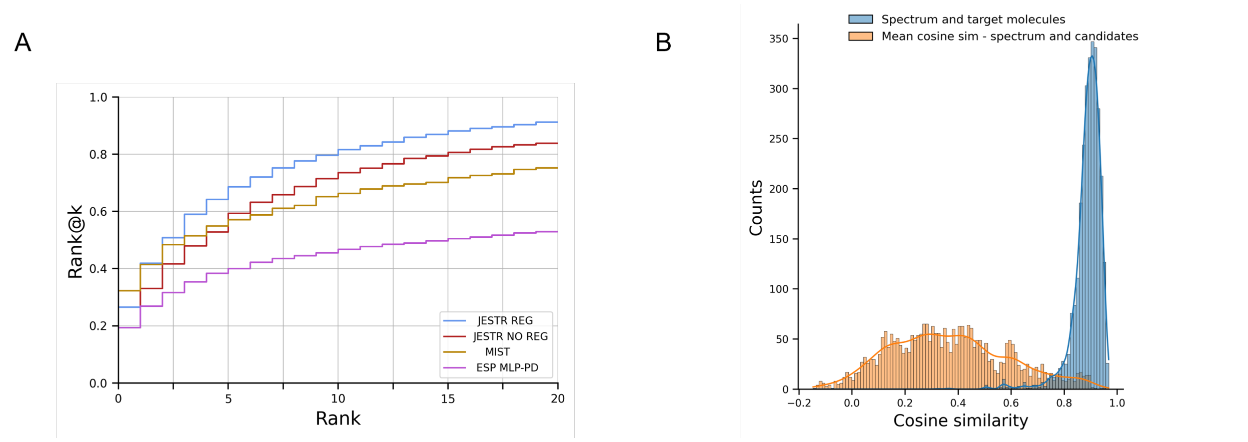

Figure S3: Results. A. Rank@k results for JESTR on MoNA, with and without regularization, MIST, and ESP MLP-PD. B. Distribution of cosine similarities of query spectra and target/candidate molecules in the MoNA test set with contrastive learning using JESTR.

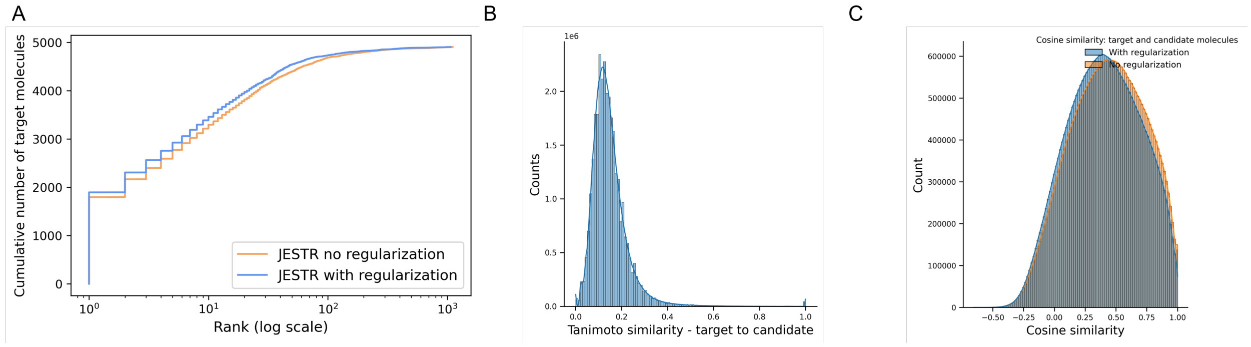

Figure S4: Regularization analysis for JESTR for NIST2020. A. Regularization improves rank@k by significantly placing more targets at rank 1. B. Distribution on Tanimoto similarities on the ECFP fingerprints between target and candidates in the training set. C. Distribution on cosine similarities, with and without regularization, of the target and candidates within the test set.

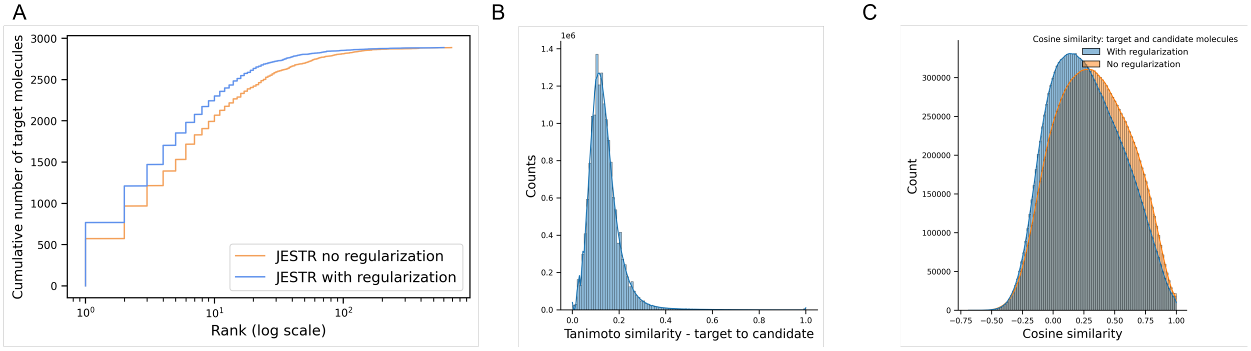

Figure S5: Regularization analysis for JESTR for MoNA. A. Regularization improves rank@k by significantly placing more targets at rank 1. B. Distribution on Tanimoto similarities on the ECFP fingerprints between target and candidates in the training set. C. Distribution on cosine similarities, with and without regularization, of the target and candidates within the test set.

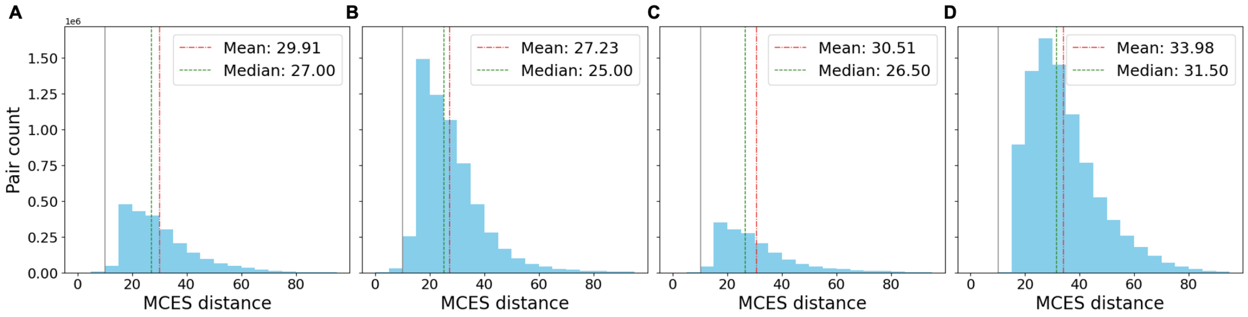

Figure S6: Distribution of MCES distances between molecules in SIRIUS's training set and test molecules from A)NPLIB1, B)NIST2020, C)MoNA, D)MassSpecGym. The solid gray line marks the MCES distance of 10.

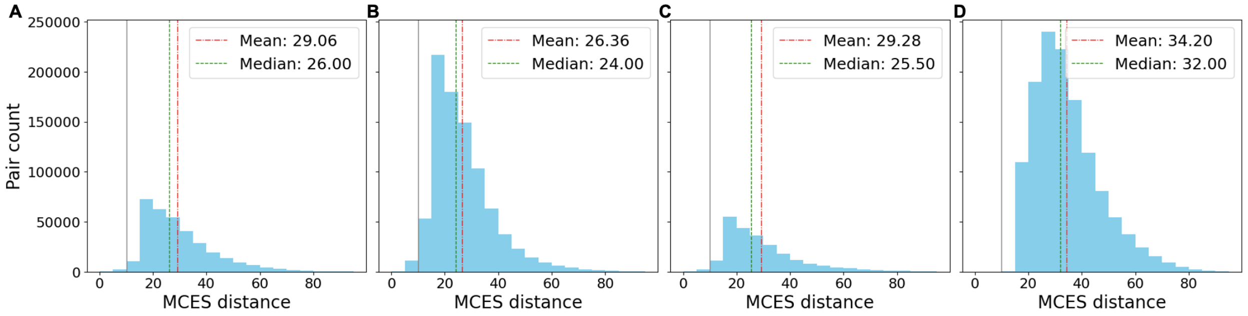

Figure S7: Distribution of MCES distances between molecules in CFM-ID's training set and test molecules from A)NPLIB1, B)NIST2020, C)MoNA, D)MassSpecGym. The solid gray line marks the MCES distance of 10.

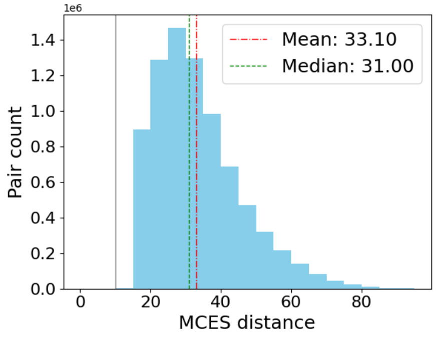

Figure S8: Distribution of MCES distances between train and test molecules in the MassSpecGym. The solid gray line marks the MCES distance of 10.
